# Microsponge based gel as a simple and valuable strategy for formulating and releasing Tazarotene in a controlled manner

**DOI:** 10.1038/s41598-022-15655-z

**Published:** 2022-07-06

**Authors:** Alaa Khattab, Abdulhakim Nattouf

**Affiliations:** Faculty of Pharmacy, Damascus University, Damascus, Syria

**Keywords:** Diseases, Nanoscience and technology

## Abstract

This study aims to deliver Tazarotene (TZR) in a controlled manner to reduce adverse effects in the form of a microsponge-based gel. It adopts the methodology of a similar study by the undersigned authors with respect to the drug Clindamycin. Under both studies, the methodology used is emulsion solvent diffusion. Accordingly, we altered the concentrations of polymer and emulsifier to generate four formulations of TZR microsponges. Additionally, we used two types of emulsifiers and two types of solvents to develop two further microsponge formulations. We then studied the physical properties of each formulation, as well as drug-polymer interactions. Echoing findings from our prior study of Clindamycin, we found that microsponge formulations coded by T1 and T3 had superior production yield and entrapment efficiency, and their particle size was suitable for dermal application. As in the prior Clindamycin study, each of the T1 and T3 microsponge formulations were incorporated into a Carbopol gel and evaluated in vitro. The optimal formulation was found to be the microsponge formulation gel T8, which released 87.63% of TZR over 12 h. No significant interactions between the drug and excipients were found through Fourier transform infrared spectroscopy and differential scanning calorimetry.

## Introduction

Tazarotene (TZR) is a novel and a unique drug that treats the most common dermatological diseases and conditions such as psoriasis, acne and photoaged/sun-damaged skin. TZR is a third-generation synthetic receptor-selective retinoid, and is available in cream, gel and foam forms^1^.

Acne vulgaris is a common inflammatory pilosebaceous condition affecting more than 85% of adolescents and young adults^2^. A complex interplay of four factors, follicular hyperkeratosis, hyperseborrhea, dysbiosis of the cutaneous microbiome and inflammation together describe the pathogenesis of acne^3,4^. Acne vulgaris manifests as inflammatory or non-inflammatory skin lesions, which in severe cases can develop into deep purulent skin lesions and cause scarring. These lesions are mostly located on the patient’s face and upper body, and thus induce psychosocial impacts such as depression, anxiety, and lower self-esteem^4,5^. Retinoids are effective first-line treatment for acne, and, according to some reports, TZR has been the most powerful retinoid^6,7^.

Psoriasis is a very common chronic autoimmune skin disease that follows a pattern of alternating periods of remission and relapse, and it is characterized by the presence of erythematous lesions or plaques covered with silvery scales that may itch, burn or bleed. These scaly plaques are mainly found on the elbows, knees, scalp and trunk, and they occur due to many factors that include hyperproliferation and abnormal differentiation of keratinocytes and inflammation. Therefore, Psoriasis considerably impacts the quality of life of patients, and many of them develop psychiatric disorders. Most psoriasis patients require topical therapy, and the main topical medications for psoriasis are corticosteroids and TZR^8^. It has been reported that TZR is as effective as corticosteroids, prolongs the remission phase and circumvents the problems of corticosteroids^9^.

When TZR is applied topically, it is rapidly subjected to a cutaneous metabolism and converted to the active form, Tazarotene acid, which is responsible for the anti-hyperproliferative and anti-inflammatory effects of the drug by binding to specific receptors and regulating the expression of particular genes. Thus, TZR plays a key role in acne and psoriasis therapy^10^.

However, formulating TZR in conventional dosage forms does not achieve the maximum therapeutic benefits of the drug due to some limitations. One of these critical limitations is the burst release of the drug, which occurs immediately upon application, leading to skin irritation. For acne and psoriasis patients who already experience psychological harm from their conditions or suffer from pruritic and painful lesions, the added irritation poses a troublesome challenge^11,12^. Another problem is that the lipophilicity of TZR restricts its incorporation into acceptable conventional formulations^1^. Based on the previous facts, patients may discontinue their treatment. Consequently, it is necessary to investigate new formulations of TZR that overcome these disadvantages.

Numerous modern drug delivery systems, such as liposomes, niosomes, solid lipid nanoparticles and microemulsions, have been studied to formulate and release poorly soluble drugs in an extended manner^13^. However, Microemulsions contain high quantities of surfactants and cosurfactants, which can cause irritation when used for long periods of time^14,15^. Similarly, solid lipid nanoparticles usually exhibit initial burst release and have low drug entrapment efficiency^16^. Besides, most of the novel formulations face stability and production problems^13,14,16^.

Microsponges are an attractive drug delivery technology, surpassing the drawbacks of the conventional formulations and the other new drug delivery systems. They are polymeric, porous microparticles consisting of a large number of tiny pores loaded with active ingredients^17^. These microspheres lodge themselves in the crevices of the skin, and gradually release appropriate concentrations of the drug at the desired site for an extended period of time without passing through the skin. Thus, microsponges would minimize the dose-dependent adverse effects of TZR, while still sustaining its efficacy. In addition, microsponges can hold a wide range of hydrophobic drugs with high entrapment efficiency and exhibit good stability^18^.

The primary objective of this research was to produce a topical formulation of TZR via using microsponge technology to control the drug delivery and consequently decrease the TZR local side effects. Microsponges were prepared by changing several factors to highlight the effect of many formulation variables on the microencapsulation efficiency. Taking into consideration the need for obtaining an aesthetic product, the optimized microsponges formula was intended to have a particle size of no larger than 30 um (to prevent any rough sensation) and to be incorporated into a light and inert vehicle base like aqueous Carbopol gel.

## Materials and methods

### Materials

Tazarotene was bought from Sigma–Aldrich (Germany). Polyvinyl Alcohol was purchased from HiMedia labs (Mumbai). Carbopol 934 was obtained from Loba chemie (India). Ethyl cellulose was purchased from Sigma-Aldrich (Germany). Tween 80 was purchased from Riedel-De häen (Seelze-Hannover, Germany). Dichloromethane, ethanol, sodium hydroxide and HPLC solvents were of analytical grade and obtained from Merck (Germany).

### Methods

#### Preparation of TZR microsponges

The microsponges containing TZR were formulated using quasi-emulsion solvent diffusion approach, similar to our earlier work on Clindamycin^12^. The approach involved two parts: an internal phase and an external phase. In order to prepare the internal phase, ethyl cellulose is dissolved in 20 mL of solvent. After obtaining a clear solution, TZR was added and dissolved via ultrasonication at 25 °C for 30 min to achieve a homogeneous clear solution. With a stirring rate of 1200 rpm, the resultant solution was gently injected into an aqueous surfactant solution (external phase). The evaporation of DCM caused the microsponges to develop after 3 h of stirring. Finally, the microsponges were filtered, washed with distilled water, and dried in a hot air oven at 40 °C for 24 h^19^. As indicated in Table 1, several formulation batches were formed.

**Table 1:** Tazarotene microsponge formulations.

Ingredients	T1	T2	T3	T4	T5	T6
Tazarotene (mg)	100	100	100	100	100	100
Ethyl cellulose (mg)	200	300	300	200	300	300
Dichloromethane (mL)	20	20	20	20	–	20
Ethanol (mL)	–	–	–	–	20	–
PVA (mg)	200	200	300	300	300	–
Tween 80 (mg)	–	–	–	–	–	300
Distilled water (mL)	150	150	150	150	150	150

#### Evaluation of microsponge

##### Production yield and entrapment efficacy

The entrapment and yield of the developed microsponges were determined using the following equations ^12,20^:

1 $$\text{Production yield \% }=\frac{weight\, of \,the\, dried \,microsponges }{sum \,of \,the\, initial \,dry \,weight\, of\, starting\, materials}\times 100,$$

2 $$\text{Loading efficacy \%}= \frac{actual \,drug \,content }{theoretical \,drug \,content }\times 100.$$

##### Determination of particle size

An optical microscope with a stage micrometer was used to quantify the mean particle size of TZR-loaded microsponges^12^.

##### Microscopic analysis

T1, T3 microsponge formulations were investigated under a polarizing microscope to assess the sphericity of the microsponges and other features.

##### Preparation of TZR microsponge gel

A gel of TZR microsponge was prepared using the same manner as a gel of Clindamycin-free base microsponge^12^. Under moderate stirring, Carbopol 934 was added to a mixture of water and glycerine. Parabens and edetate disodium were dissolved in water and mixed with the prior mixture. After that, this mixture was neutralized by adding triethanolamine with gentle mixing. Finally, TZR microsponges (equivalent to 0.1% w/w of TZR) were incorporated to obtain homogenous TZR microsponge-loaded gels. Table 2 displays TZR microsponge gel compositions.

**Table 2:** TZR microsponge-loaded gel formulations.

Ingredients	T7	T8
Microsponges (mg)	T1 formula equivalent to 100 mg of drug	T3 formula equivalent to 100 mg of drug
Carbopol 934 (g)	0.3	0.3
Glycerine (g)	5	5
Triethanolamine	q.s	q.s
Methyl paraben (g)	0.18	0.18
Propyl paraben (g)	0.02	0.02
EDTA (g)	0.05	0.05
Distilled water (g)	q.s.100	q.s.100

#### Gel evaluation

##### Physical appearance

The colour, appearance, and consistency of the prepared gels were assessed visually^12^.

##### pH determination

The pH of the gel formulations was calculated using a digital pH meter (HANNA 211)^12^.

##### Viscosity determination

The viscosity of the gel formulations was assessed by a digital viscometer (Rotary viscometer STS-2011) with spindle R7 at 25 ± 10 °C^12^.

##### Drug content determination

One gram of each gel formulation was mixed with 100 mL of ethanol 95% to determine the TZR content in the gel. The solution was filtered and spectrophotometrically analysed at 351 nm using a UV–Vis spectrophotometer with suitable sample dilution^12^.

##### In vitro drug release tests

A vertical Franz diffusion cell with a reservoir capacity of 9.5 mL was used for in vitro release investigations. Between the compartments of the diffusion cell, a cellulose nitrate membrane with an effective diffusion area of 2.54 cm^2^ was inserted. The receptor medium was composed of a mixture of water and ethanol (50:50, v/v), and it was kept at 32 ± 0.1 °C and swirled constantly. Each formulation weighing 0.5 g of microsponge-based gel was placed on the donor side. 2 mL of the sample was taken from the receiver fluid and replaced with an equal volume of fresh receptor fluid at predefined time intervals. Collected samples were assayed by an UV–Vis spectrophotometer after a suitable dilution^12,21–23^.

##### Drug release kinetics determination

The release data was analysed using zero order, first order, Higuchi, Hixson-Crowell, and Korsmeyer-Peppas to explore the pattern of TZR release from the microsponge loaded gels^12^.

##### Stability testing

Formulation T8 was subjected to stability studies according to ICH guidelines. The formulations were monitored for up to 6 months at 40 ± 2°/75 ± 5%. At 1, 2, 3, 4, 5, and 6 months, the appearance, pH, viscosity, drug content, and drug release of the gel were evaluated^12,21^.

#### Compatibility studies

##### Differential scanning colorimetry (DSC)

DSC studies were done on pure TZR and T3 microsponge formulation (DSC131, SETARAM, France). The samples were accurately weighed and packed in aluminium pans. In a nitrogen environment, samples were heated at a rate of 10 °C/minute throughout a temperature range of 25–450 °C^12,24^.

##### Fourier transformer infrared spectroscopy (FTIR)

FTIR spectra of pure TZR and T3 microsponge formulation were recorded using KBr disc to check compatibility.(Bruker IR, Germany)^12,25^.

### Statistical analysis

All values are the average of three experiments. Using Microsoft Excel 2010, the data was analysed by Student’s t-test, with P < 0.05 deemed significant^12^.

## Results and discussion

### Determination of production yield% (PY%) and entrapment efficacy% (EE%) of the microsponge formulations

**Figure 1:**
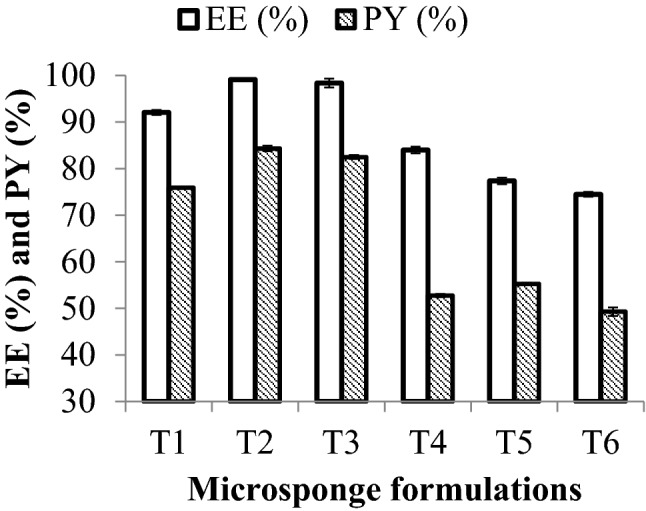
Graph displaying PY (%) and EE (%). Values are expressed as mean ± SD (n = 3).

Figure 1 and Table 3 show the values of EE% and PY% of TZR-loaded microsponges. Microsponge formulations coded by T1, T2, T3,T4 were prepared as microsponge formulations loaded with Clindamycin free base and coded by C1,C2, C3, C4 respectively in our previous work^12^. However, microsponge formulations loaded with TZR (T1, T2, T3,T4) gave significantly higher EE% and PY% than microsponge formulations loaded with Clindamycin free base (C1,C2, C3, C4) respectively. This can be attributed to the extremely low solubility of TZR in the external aqueous phase during the microsponge preparation. As shown in Table 3, it can be concluded that for the same amount of PVA, adding more EC during the preparation of microsponges dramatically enhanced the EE% and PY% of the microsponges. In contrast, using more PVA with a fixed concentration of EC decreased the EE% and PY% of the prepared microsponges. We could explain these results by showing how at a higher level of EC, a larger amount of this polymer surrounded the drug units, resulting in encapsulating a larger amount of the drug^26^. On the other hand, using the low level of PVA (which is a nonionic emulsifier) decreased the solubility of TZR in the outer aqueous phase and hence minimized its loss^27^. Microsponge prepared with DCM (T3) gave higher EE% and PY% than that of ethanol (T5), as illustrated in Table 3. DCM was poorly miscible with water, which led to the slow diffusion of the solvent out of the emulsion droplets to the external aqueous medium, which resulted in slow precipitation of the polymer matrix, thus increasing the chance for entrapping the drug inside the polymer. The formulation prepared using PVA as an emulsifier (T3) was found to show higher PY%, LD% than the formulation prepared using tween 80 (T6). This may be due to the lower solubility of TZR in PVA than in tween 80, and the drug was therefore retained in the internal phase when microsponges were prepared with PVA^28^.

**Table 3:** EE% and PY% results of TZR microsponge formulations.

Formula	EE % ± S.D.	PY % ± S.D.
T1	92.06 ± 0.02	75.91 ± 0.23
T2	99.1 ± 0.13	84.32 ± 0.08
T3	98.37 ± 0.05	82.48 ± 0.42
T4	83.99 ± 0.7	52.74 ± 0.35
T5	77.36 ± 0.7	55.25 ± 0.2
T6	74.48 ± 0.5	49.28 ± 0.1

### Particle size

As shown in Table 4, increasing the concentration of EC produces microsponges with a larger size. We could explain this by the fact that preparing the microsponges with a higher level of EC caused an augmentation in the viscosity of the internal organic phase, which could in turn restrict the division of the globules into smaller particles^29^. Conversely, increasing the concentration of PVA produces microsponges with a smaller size. This was most probably due to the stability enhancement of the initial emulsion, which prevented droplets from merging, and thus helped the formation of finer microparticles^30^.

**Table 4:** Particle size results of TZR microsponge formulations.

Formula	Particle size (µm) ± S.D.
T1	25.91 ± 0.13
T2	37.25 ± 0.53
T3	28.61 ± 1.02
T4	20.34 ± 1.12

### Selection of optimal microsponge formulations

T1 and T3 formulations were chosen for further evaluation, since they had high EE% and PY% and their particle size was smaller than 30 µm.

### Microscopic analysis

**Figure 2:**
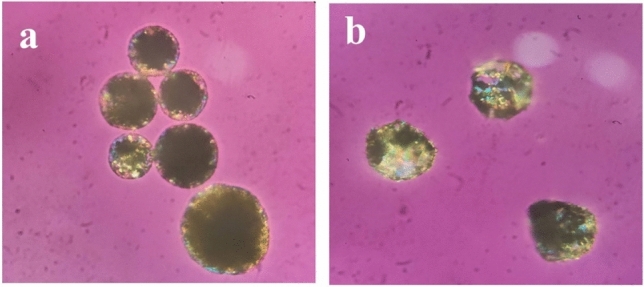
Microscopic images of (**a**) T3 formulation, 100×, (**b**) T1 formulation, 100×.

Investigations by the polarized light microscope (Fig. 2) showed the spherical shape of the microsponges, which would facilitate their incorporation into the gel formulation. Besides, there were no free drug crystals found. This suggests that all drug units were entrapped inside the microsponges. DSC studies confirmed the results noted above.

### Physical properties and appearance of gels

T7 and T8 gel formulations had a smooth appearance and acceptable values of viscosity, pH and drug content. They were viscous, spread easily and had a pH suitable for dermal application. In addition, the results of the drug content test revealed that TZR was homogeneously incorporated in both gel formulations. Data for viscosity, pH and drug content are listed in Table 5.

**Table 5:** Physical properties and appearance results of TZR gels.

Formula	PH ± S.D	Viscosity (cps) at 25 °C	Drug content (%) ± S.D.
T7	6.5 ± 0.03	24,110 ± 22	98.35 ± 0.1
T8	6.2 ± 0.08	23,914 ± 36	99.13 ± 0.02

### In vitro drug release tests

**Figure 3:**
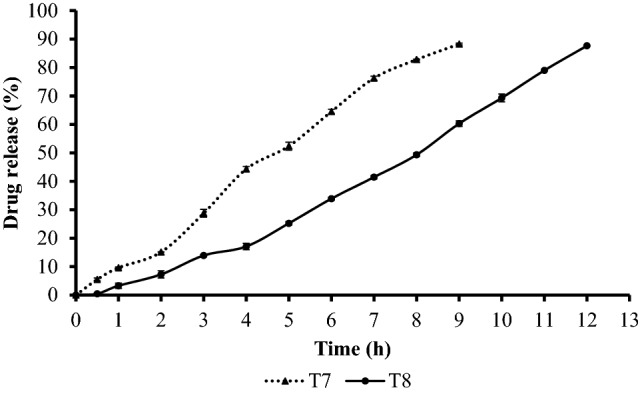
TZR release percentage from T7 and T8 formulations against time (h). Values are expressed as mean ± SD (n = 3).

Figure 3 shows that the T8 formulation (which contains 300 mg EC) released 87.63% of TZR over 12 h, whereas the T7 formulation (which contains 200 mg EC) released 88.24% of TZR over 9 h. Therefore, it could be concluded that the levels of EC clearly affected the extent of drug release. Consistent with findings from our prior study of Clindamycin, microsponges with a higher proportion of EC fabricated larger microsponges. Thus, the drug needed more time to diffuse through them and release into the dissolution medium^12,31^. In addition, as with the clindamycin study, no initial burst release was found in the two gel formulations. This indicates the absence of non-encapsulated TZR^25^. Microscopic analysis and DSC studies results were in accordance with the previous finding.

### Drug release kinetics determination

The release of the drug from T7 and T8 gel formulations followed zero order kinetics, since the curve represented by the model equation had the highest linearity (Table 6). The zero order kinetics explains the controlled release of the prepared microsponge gels over time. The value of n was greater than 0.89 for both gel formulations, suggesting that the TZR release mechanism from T7 and T8 gel formulations was super case II transport^32^.

**Table 6:** Release kinetics results of T7 and T8 formulations.

Formula	Zero	First	Highochi	Peppas		Hicxon
	R^2^	R^2^	R^2^	R^2^	n	R^2^
T7	0.990	0.957	0.968	0.987	1.02	0.943
T8	0.985	0.871	0.913	0.983	1.52	0.950

### Stability testing

As listed in the Table 7, the clarity, pH, drug content and drug release percentage of T8 gel formulation did not alter significantly during the stability testing. Furthermore, the drug release profiles of this formulation were compared prior and after a 6-month stability trial to estimate the similarity factor (f2). The value of the similarity factor was f2 = 77.79 (> 50), indicating that the T8gel formulation was quite stable^33^.

**Table 7:** Stability testing results of T8 formulation.

Time (months)	Physical appearance	PH ± S.D.	Drug content (%) ± S.D.	CDR (%) ± S.D.
0	–	6.2 ± 0.27	99.13 ± 0.02	87.63 ± 0.02
1	No alteration	6.1 ± 0.51	99.01 ± 0.91	86.35 ± 0.61
2	No alteration	6.1 ± 0.07	98.74 ± 0.07	87.24 ± 0.07
3	No alteration	6.2 ± 0.91	97.91 ± 0.49	86.19 ± 0.91
4	No alteration	6.3 ± 0.3	97.35 ± 0.15	85.6 ± 0.37
5	No alteration	6.1 ± 0.72	96.93 ± 0.07	85.38 ± 0.16
6	No alteration	6.2 ± 0.42	96.07 ± 0.09	86.31 ± 0.18

### Compatibility studies

#### Differential scanning colorimetry (DSC)

**Figure 4:**
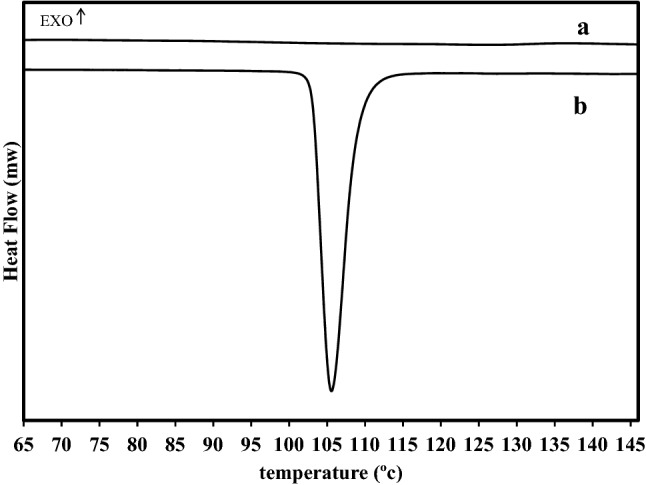
DSC charts of (**a**) T3 formulation, (**b**) pure TZR.

DSC analysis was performed to study the physical properties of TZR and TZR-loaded microsponges and verify the drug compatibility with excipients. A sharp endothermic peak at 105.61 °C was observed on a DSC thermogram of pure TZR (Fig. 4b), indicating the drug’s melting temperature and reflecting its purity. However, this peak was not found on the thermogram of TZR microsponge formulation T3 (Fig. 4a). Thus, it could be asserted that the drug was distributed uniformly into the microsponges, which, like all cross-linked polymers, had an amorphous structure^34,35^.

### Fourier transform infrared spectroscopy FTIR

**Figure 5:**
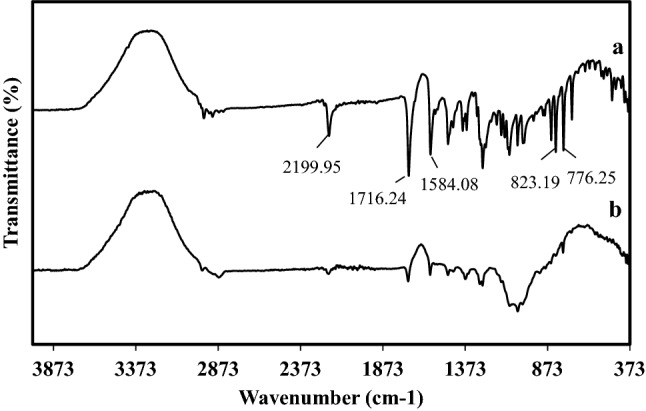
FTIR spectrums of (**a**) pure TZR, (**b**) T3 formulation.

Figure 5a,b exhibit the infrared spectrum of pure TZR and T3 microsponge formulation, respectively. TZR spectrum shows absorption bands at 2199.95 cm^−1^ for disubstituted alkyne C≡C stretching, at 1584.08 cm^−1^ for C=N stretching, and at 1716.24 cm^−1^ due to carbonyl group stretching of the ester. Other peaks were also at 823.19 and 776.25 cm^−1^, which reflect bending of aromatic C–H. These results were in agreement with those found in the literature^36–38^. According to the spectrum of T3 microsponge formulation, the intensity of most TZR characteristic peaks has diminished, and some peaks have vanished. This could be attributed to the restriction within the polymer matrix, in line with our findings in the Clindamycin study. In addition, no new peak was identified in the FTIR spectra of T3 microsponge formulation, indicating that there was no probable interaction between TZR and other formulation ingredients^12,36^.

## Conclusions

The purpose of this work was to formulate a TZR-based microsponge gel with acceptable physicochemical properties and sustained release for improved drug tolerance. Six formulations of TZR-loaded microsponges, T1, T2, T3, T4, T5 and T6, were produced using the quasi emulsion solvent diffusion method with two types of solvents (ethanol and DCM), two types of surfactants (tween 80 and PVA), two concentrations of polymer EC (200 mg and 300 mg) and two concentrations of surfactant PVA (200 mg and 300 mg). These microsponges were then analysed for production yield, entrapment efficacy and particle size. T1 and T3 formulations showed high yield and entrapment efficacy, and they achieved a particle size less than 30 µm, making them appropriate for cutaneous application.

In addition, no free drug crystals were observed in these formulations and their microsponges had a spherical shape as shown by microscopic analysis. Therefore, T1 and T3 formulations were incorporated into Carbopol gel to formulate T7 and T8 formulations, respectively. Then T7 and T8 formulations were in vitro evaluated. The appearance, drug content, viscosity and pH of the T7 and T8 formulations were all acceptable. Additionally, both of these formulations prolonged the release of the drug without causing an initial burst release. However, the T8 formulation outperformed the T7 formulation by releasing the medication for up to 12 h. This formulation was stable after a six-month stability trial and showed good compatibility between the drug and excipients through FTIR and DSC studies. Thus, TZR-loaded microsponge based gel was found to be promising as a controlled release drug delivery system for overcoming the side effects caused by the conventional TZR formulations. As a result, it could improve the patients’ adherence to acne and psoriasis treatment and might serve as a worthwhile contribution to the pharmaceutical market in the near future. On the other hand, TZR in the present study was prepared nearly identically as CLN had been prepared in our earlier study. Hence, this present study, along with our previous study, both open the door for further development of microsponge based gel of a TZR/CLN fixed combination for acne treatment. This strategy would help to incorporate a hydrophilic drug (CLN) and a lipophilic drug (TZR) into a single dosage form via a simple and cost-effective method.

## Data Availability

All data generated or analysed during this study are available within the article.
